# *QuickStats*: Percentage* of Adults Aged ≥18 Years with Serious Psychological Distress in the Past 30 Days,^†^ by Sex and Age Group — National Health Interview Survey,^§^ United States, 2021

**DOI:** 10.15585/mmwr.mm7208a5

**Published:** 2023-02-24

**Figure:**
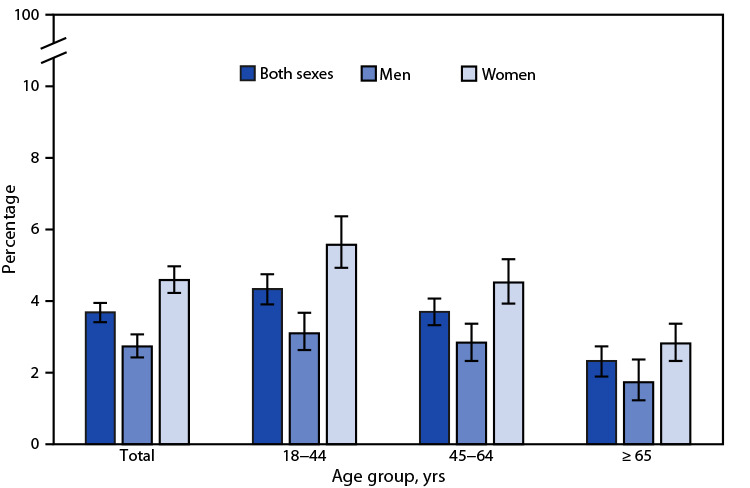
In 2021, 3.7% of adults aged ≥18 years had serious psychological distress in the past 30 days with percentages higher among women (4.6%) than among men (2.7%). The higher percentages among women were seen across all age groups: 5.6% versus 3.1% in adults aged 18–44 years, 4.5% versus 2.8% in those aged 45–64 years, and 2.8% versus 1.7% in those aged ≥65 years. The percentage of women who had serious psychological distress in the past 30 days decreased with age; the percentage of men who had serious psychological distress in the past 30 days was higher among those aged 18–44 and 45–64 years than among those aged ≥65 years.

**Source:** National Center for Health Statistics, National Health Interview Survey, 2021. https://www.cdc.gov/nchs/nhis.htm

